# Correlation of ferritin and glutathione peroxidase 4 (GPX4) level as a marker of ferroptosis process in endometrioma

**DOI:** 10.1038/s41598-024-85017-4

**Published:** 2025-02-05

**Authors:** Rina Nulianti, Hartanto Bayuaji, Mulyanusa Amarullah Ritonga, Tono Djuwantono, Dian Tjahyadi, Anita Rachmawati, Sri Ratna Dwiningsih, Aisyah Shofiatun Nisa, Putri Nadhira Adinda Adriansyah

**Affiliations:** 1 Department of Obstetrics and Gynecology, Faculty of Medicine, Universitas Padjadjaran – Dr. Hasan Sadikin General Hospital, Jalan Pasteur no 38 Bandung, Bandung, 40161 Indonesia; 2 Department of Obstetrics and Gynecology, Faculty of Medicine, Dr. Sutomo General Hospital, Universitas Airlangga, Bandung, Indonesia

**Keywords:** Endometrioma, Ferritin, Glutathione peroxidase 4 (GPx4), Diseases, Reproductive disorders

## Abstract

Endometriosis is a chronic inflammatory disease characterized by the presence of endometrial-like tissues (glands and stroma) located outside the uterine cavity. The pathophysiology of this condition remains incompletely understood. Local bleeding and inflammation within endometriosis lesions, which occur in an environment rich in iron, reactive oxygen species (ROS), and free radicals, can disturb the balance of iron within the peritoneal cavity. This disruption can trigger oxidative injury and an inflammatory response, leading to ferroptosis, particularly in the endometrioma phenotype. This research utilized an observational analytical method with a cross-sectional design to examine the relationship between ferritin and GPx4 levels by assessing them simultaneously. The data collection occurred at Dr. Hasan Sadikin Hospital, Cibabat Hospital, Bandung Kiwari Hospital, and Limijati Women and Children Hospital Bandung. Observational analytic data were gathered between February and July 2023 from female patients diagnosed with endometrioma who underwent either laparoscopic or laparotomy surgery, either for therapeutic or diagnostic reasons. There were 58 patients who met the inclusion and exclusion criteria in this study. A significant correlation was observed between ferritin and glutathione peroxidase 4 (GPx4) levels with a correlation coefficient of -0.600 (*p* < 0.001). However, there was no significant correlation between both ferritin levels and the severity of endometriosis (based on Association of Gynecologic Laparoscopists staging). There was significant correlation between ferritin and glutathione peroxidase 4 (GPx4) levels.

## Introduction

Endometriosis is a chronic inflammatory disease characterized by the presence of endometrial-like tissues (glands and stroma) located outside the uterine cavity affect approximately 10–15% of reproductive age^1,2^. This condition is often associated with symptoms such as pain and infertility, significantly impacting the affected women’s quality of life.3 The incidence of endometriosis among women with chronic pelvic pain is approximately 70‒75%, whereas among those experiencing infertility, it ranges from 25 to 40%^3^. Dr. Cipto Mangunkusumo Hospital reported complaints of chronic pelvic pain around 82.5% of cases, dysmenorrhea 81%, and infertility 33.7%^4^.

Patients with endometriosis commonly present with symptoms such as dysmenorrhea, cyclic pelvic pain, dyspareunia, dyschezia, dysuria, infertility, or may even be asymptomatic^4,5^. The exact pathophysiology of endometriosis remains unclear, although various factors are believed to play a role, including retrograde menstruation, hormonal and immunological influences, stem cells, genetic/epigenetic factors, celomic metaplasia, remnants of Mullerian ducts, and lymphatic and vascular spread^6–8^. Endometriosis manifests in three main phenotypes: superficial endometriosis, endometrioma, and deep infiltrating endometriosis (DIE). Endometriomas, ovarian cysts covered with endometrial tissue, histologically resemble eutopic endometrium and contain significantly higher concentrations of free iron, reactive oxygen species (ROS), and lipid peroxide compared to other benign cysts^5,9,10^.

Excessive iron in the peritoneal fluid can lead to oxidative damage and an inflammatory response. Normally, proteins like ferritin and transferrin help regulate iron radicals and oxygen to prevent harmful reactions, but this balance is disrupted in pathological conditions^11,12^. When the production of reactive oxygen species (ROS) exceeds the body’s antioxidant defenses, oxidative stress occurs, impacting various cellular processes, including programmed cell death^11^. Ferroptosis is a form of programmed cell death distinct from apoptosis, is characterized by iron-catalyzed lipid peroxidation through both non-enzymatic (Fenton reaction) and enzymatic mechanisms (lipooxygenase)^7,8,13^.

Several ideas about the aetiology of endometriosis exist, including retrograde menstruation, chemotaxis of corpora cavernosa, lymphatic and vascular dissemination, hormonal influences, immunological factors, and extrauterine stem cell differentiation. The most acknowledged and credible explanation is the “retrograde menstruation” posited by J.A. Sampson in 1927, which involves the backwards transport of erythrocytes and endometrial tissue from menstrual blood through the fallopian tubes, leading to implantation in the peritoneum and resulting in inflammation, immune dysfunction, and oxidative stress^14^. Despite its benign nature, endometriosis has many characteristics of malignant neoplasms to malignant neoplasms, including local invasion, resistance to apoptosis, and angiogenesis^15^.

Oxidative stress refers to the imbalance between reactive oxygen species and antioxidants, which mostly leads to cell death through mechanisms such as apoptosis, autophagy, and ferroptosis^16,17^. Apoptosis is involved in follicular atresia and the cyclical shedding of the endometrium. Furthermore, the death of endometrial cells may facilitate the proliferation of fallopian tube cells in ectopic sites during menstrual shedding and may be linked to the development of endometriosis^18^.

Ferroptosis is an innovative form of cell death driven by iron, characterised by cytoarchitectural damage resulting from lipid peroxidation^19^. Ferroptosis is intricately linked to the aetiology of cancer, ischemia-reperfusion injury, ischemic organ damage, neurological disorders, stroke, and renal failure^20^. The precise mechanism of epithelial-mesenchymal transition (EMT) remains unidentified; however, recent research indicates that EMT is likely associated with iron-dependent oxidative stress-mediated ferroptosis^21,22^.

In endometrioma, iron can induce ROS production through auto-oxidation and the Fenton reaction. Several markers, including morphological, genetic, biochemical, and protein markers like ferritin and GPx4, can indicate ferroptosis^13^. Endometriosis still considered as multifaceted gynaecological disorder with an ambiguous aetiology, can adversely impact quality of life^23^. Currently, histological confirmation is the definitive criterion for diagnosis, while noninvasive biomarkers have proven to be unreliable for practical use^24^.

This study aims to investigate the correlation between ferritin and glutathione peroxidase 4 (GPx4) levels as markers of ferroptosis in endometriomas. Understanding the roles of ferritin and GPx4 in the pathophysiology of endometriosis tissue can potentially lead to therapeutic strategies targeting cell death pathways. Parameters examined include ferritin and GPx4 levels, as well as the stage of endometriosis according to the Association of Gynecologic Laparoscopists (AAGL) classification^25^.

## Materials and methods

This study aimed to analyze the correlation between ferritin and glutathione peroxidase 4 (GPx4) levels as markers of ferroptosis in endometriomas. Conducted as an observational analytic study with a cross-sectional, retrospective design, this research analyzed data from previously collected tissue samples and clinical records. Ethical clearance was obtained from the Health Research Ethics Committee of Universitas Padjadjaran, registered under LB.02.01/X.6.5/168/2023, and was carried out in accordance with the Declaration of Helsinki. Patients provided written informed consent for the use of their tissue samples in this research. All data was registered at the research registry under Research Registry UIN researchregistry10061.

The subjects of this study were all patients with endometrioma who underwent laparoscopic/laparotomy surgery at Dr. Hasan Sadikin Hospital, Cibabat Hospital, Bandung Kiwari Hospital and Limijati Women and Children Hospital Bandung from February-July 2023. The inclusion criteria of this study were patients with endometrioma, aged 20–40 years, patients diagnosed with endometrioma through transvaginal ultrasound and histophatology report, undergone laparoscopic/laparotomy surgery for endometrioma. While the exclusion criteria in this study were patients with history of pelvic inflammatory disease, history of pelvic malignancy, history of immune, endocrine and metabolic diseases, patients with anemia and not undergone iron supplements in the last 3 months, not using hormone drugs in the last 3 months.

Research subjects who met the inclusion and exclusion criteria were given informed consent. Tissue samples were collected from patients who underwent laparoscopic or laparotomy surgery and were suspected to have endometrioma based on preoperative ultrasound findings. During surgery, a small sample (1 × 1 cm, approximately ± 0.14 g) was taken from the lining of the suspected endometrioma. All samples were first sent for histopathological examination to confirm the diagnosis. Only samples confirmed as endometriomas by histopathology were subsequently processed for this study.

Following histopathological confirmation, each confirmed endometrioma tissue sample was prepared by cutting into small pieces and placing them into a bead tube. The tissue was dissolved in 1 ml of PBS (1X, pH 7) and then disrupted using a magnalyser. An equal volume of RIPA solution (Sigma-Aldrich, Cat. No. R0278) was added, and the sample was incubated at room temperature for 30 min. It was then centrifuged at 3000 rpm for 10 min, after which the supernatant was collected and divided into aliquots (500–1000 µl). Aliquots were stored at −80 °C for long-term storage (up to 6 months) and − 20 °C for short-term storage (up to 1 month).

Ferritin and GPx4 levels in the endometrioma tissue samples were subsequently analyzed using ELISA kits. For GPx4, the Human Glutathione Peroxidase 4, GPX4, ELISA KIT|Bioenzy|BZ-08127886-EB was used, and for ferritin, the Human Ferritin, FE ELISA KIT|Bioenzy|BZ-08122071-EB was utilized. The analyses were performed at the Molecular Genetics Laboratory, Pamitran Building, Padjadjaran University, Bandung.

The primary outcome of this study is to investigate the relationship between GPx4 and ferritin levels in patients with endometrioma. The secondary outcome of this study was to examine the comparison between GPx4 levels and various subject characteristics, including age, BMI, hemoglobin (Hb) levels, and AAGL staging of endometrioma.

The data collected was inputted and analyzed, leading to the creation of an analysis report on the research findings. The sampling method employed was consecutive admissions, where patients were included based on the order of arrival until reaching the minimum sample size. The normality of the data was assessed using the Kolmogorov-Smirnov test. To analyze the correlation between ferritin and GPx4 in the research group, Pearson correlation calculations were utilized if the data followed a normal distribution, while Spearman rank correlation calculations were used if the data were not normally distributed. The interpretation of hypothesis test results was based on the strength and direction of the correlation, as well as the p-value. A p-value less than 0.05 was considered statistically significant, indicating significance, while a p-value greater than 0.05 was deemed not significant. The data was recorded in a specialized form and processed using the SPSS Statistics 26 for Mac program, maintaining a confidence level of 95% with a significance threshold of *p* < 0.05.

## Results

### Characteristics of the sample

Table 1 presents the characteristics of research subjects in the form of age, body mass index (BMI), hemoglobin level (Hb) and endometriosis stage based on the American Association of Gynecologic Laparoscopists (AAGL) in the endometrioma group. It can be seen that the ages of the research subjects who participated in the study were 20–24 years old with a total of 6 people (10.9%), 16 people aged 25–29 years (29.1%), 12 people aged 30–34 years (21, 8%), and the largest composition was mainly around 35–40 years with a total of 21 people (38.2%) with mean (SD): 31.7 (5.6).

**Table 1:** Characteristics of research subjects.

Characteristics	Number	%	Mean ± SD
Age (years old):			
20–24	6	10,9	
25–29	16	29,1	
30–34	12	21,8	
35–40	21	38,2	
Average			31.7 ± 5.6
Body mass index (kg/m^2^)			
< 18,5 (*underweight*)	4	7,3	
18,5–22,9 (normal)	31	56,4	
23–24,9 (*overweight)*	16	29,1	
≥ 25 (*obese*)	4	7,3	
Hb (g/dl):			
12–13,9	43	78,2	
14–16	12	21,8	
Average			12.14 ± 0.94
AAGL staging:			
I	3	5,5	
II	10	18,2	
III	13	23,6	
IV	29	52,7	
Average			22.0 ± 9.88

The body mass index levels were distributed in the categories of thin, 4 people (7.3%), normal 31 people (56.4%), overweight 16 people (29.1%) and obese 4 people (7.3%). Most composition in the normal category. Blood hemoglobin levels were most distributed at hemoglobin levels of 12–13.9 g/dl amounting to 43 people (78.2%), while hemoglobin levels of 14–16 g/dl amounted to 12 people (21.8%) with an average (SD) 12.14 (0.94).

Based on the AAGL stage of the operation, it was found that stage I had 3 people (5.5%), stage II had 10 people (18.2%), stage III had 13 people (23.6%), and the highest composition was in stage IV there were 29 people (52.7%) with mean (SD): 22.0 (9.88). The composition of the research subjects, because they only came from the endometrioma group, was homogeneous.

Table 2 presents descriptive research subjects based on the variables studied, namely ferritin and GPx4 levels. A data normality test was carried out to determine whether the data was normally distributed or was not normally distributed. The data normality test used the Kolmogorov-Smirnov test because the number of samples was > 50. From the results of the normality test it was obtained that the GPx4 variable had a p value > 0.05, meaning that the data was normally distributed, while for ferritin levels the value was obtained *p* < 0.001 meaning the data was not normally distributed.

**Table 2:** Descriptive statistics of examination results for GPx4 and ferritin level.

Statistical measure					
Variabel	Average	SD	Median	Range	Data normality test (p* value)
GPx4 level (U/g)	4,238	0,831	4,3	2,77−6,55	0,450
Ferritin level (mcg/L)	106,20	16,70	99,36	90,54–147,97	< 0,001

*For categorical data use the Chi square test, while for numerical data use the t-test or Mann-Whitney test if the data is not normally distributed.

**statistically significant value, *p* < 0.05.

**Fig. 1:**
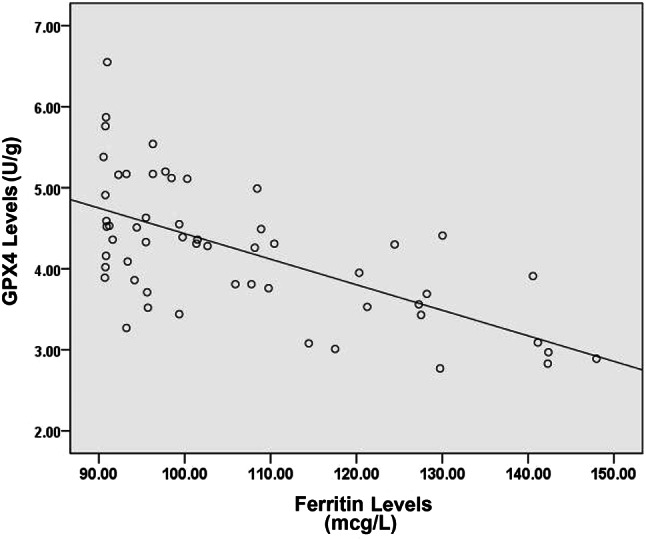
Correlation between ferritin levels and GPx4 levels in endometrioma patients.

Table 3 displays the results of statistical tests using the spearman correlation test at a 95% confidence level to show a correlation between ferritin and GPx4 levels in tissue endometrioma. The results of the correlation analysis of the various variables studied showed that there was a significant correlation between GPx4 levels and ferritin levels with *r* = -0.600 (*p* < 0.001). In endometrioma patients, the higher the ferritin level, the lower the GPx4 level. The correlation between ferritin and GPx4 levels is shown in (Fig. 1).

**Table 3:** Correlation analysis between GPx4 levels, ferritin levels, and subject characteristics in endometrioma patients.

Correlation	Correlation coefisien	
	(r)	P value
GPx4 level with age	−0,095	0,490
GPx level with BMI	−0,087	0,529
GPx level with Hb	−0,087	0,529
GPx level with AAGL staging	−0,088	0,523
GPx level with Ferritin level	**−0**,**600**	**< 0**,001**
Ferritin level with age	−0,068	0,624
Ferritin level with BMI	−0,024	0,862
Ferritin level with Hb	0,106	0,440
Ferritin level with AAGL staging	0,171	0,213

r = rank Spearman correlation.

**p < 0.05 means significant or statistically significant.

Furthermore, to determine differences in GPx4 levels and ferritin levels based on the characteristics of endometrioma patients, calculations were carried out using the Kruskal-wallis test to compare more than two groups, and the Mann-Whitney test to compare two groups. The results of the different test calculations are shown in (Table 4).

**Table 4:** Comparison of GPx4 levels and ferritin levels based on characteristics.

GPx4 level (/g)			Ferritin level (mcg/L)	
Characteristics	Median (range)	P-value	Median (range)	P-value
Age (years old):		0,587		0,880
20–24	4,80 (3,53−6,55)		103,09 (90,97–121,26)	
25–29	4,35 (2,89−5,87)		100,56 (90,70–147,97)	
30–34	4,06 (2,77−5,76)		97,53 (90,54–142,29)	
35–40	4,26 (3,09−5,20)		97,75 (90,75–141,14)	
BMI (kg/m^2^):		0,299		0,906
< 18,5	4,80 (4,39−6,55)		100,02 (90,97–108,90)	
18,5–22,9	4,26 (2,89−5,87)		99,36 (90,70–147,97)	
23–24,9	4,13 (2,77−5,38)		103,56 (90,54–142,29)	
≥ 25	4,11 (3,43−4,51)		94,28 (91,61–127,53)	
Hb (g/dl):		0,839		0,976
12–13,9	4,28 (2,77−6,55)		99,36 (90,70–142,37)	
14–16	4,45 (2,83–5,38)		99,56 (90,54–147,97)	
AAGL staging:		0,850		0,296
I	4,51 (3,56−4,55)		99,36 (94,41–127,27)	
II	4,33 (2,83−5,76)		97,42 (90,76–142,29)	
III	4,28 (3,08−5,87)		96,29 (90,70–124,46)	
V	4,09 (2,77−6,55)		100,29 (90,54–147,97)	

The p value is calculated using the Kruskal-wallis test; except for Hb levels using the Mann-Whitney test.

Table 4 shows the various characteristics studied at GPx4 levels and ferritin levels, none of which showed statistically significant differences (*p* > 0.05). The correlation of GPx4 with ferritin levels was negative (*r* = -0.600); it means that the higher the ferritin level, the lower the GPx4 level or vice versa, the lower the ferritin level, the higher the GPx level. From the picture, the hypothesis framework is accepted because the correlation is negative. Based on the correlation criteria according to Guilford: *r* < 0.20: Very weak correlation; r between 0.2 and 0.4 weak correlation; r between 0.4 and 0.7 strong correlation; r between 0.7 and 0.9 very strong correlation; r 0.9-1.0 very strong correlation. In this study, there was a strong correlation between ferritin and GPx4 levels in endometrioma tissue.

## Discussion

### Characteristics of research subjects

The characteristics of the sample included age, body mass index (BMI), hemoglobin level (Hb) and endometriosis stage based on the American Association of Gynecologic Laparoscopists (AAGL) in the endometrioma group. The research subjects who participated in this study were mainly aged 35–40 years with a total of 21 people (38.2%). This is in accordance with previous research which stated that the incidence and prevalence of endometriomas is higher in reproductive age (10–15%)^3,11,26^. The distribution of endometriomas also depends on ethnic, socioeconomic and cultural factors^27^.

The majority of study subjects had a normal BMI, comprising 31 individuals (56.4%). While epidemiological studies often suggest that individuals with endometriosis tend to have a lean BMI, findings from Holdsworth et al. (2018) contradict this, indicating that the highest BMI among women with endometriosis was within the normal range (56%), followed by overweight (25.2%), obesity (14.3%), and lean (4.5%) categories. The low distribution of obesity (7%) in this study might be attributed to its association with hyperestrogenic conditions, which can disrupt normal ovulation, leading to oligomenorrhea and amenorrhea, thereby reducing the frequency of menstruation and potentially decreasing the formation of endometriosis and endometrioma lesions^28^.

Regarding hemoglobin levels, the highest distribution was observed in the range of 12–13.9 g/dl, with 43 participants (78.2%), while 12 participants (21.8%) fell within the range of 14–16 g/dl, with an average of 12.14 (0.94) g/dl. These levels are considered normal according to the World Health Organization (WHO), which defines normal hemoglobin levels for adult women as ≥ 12 g/dl. Hemoglobin, heme, and iron derivatives play roles in maintaining the oxidant/antioxidant balance in the pathogenesis of malignant transformation of endometriosis. These components are produced through erythrocyte hemolysis and accumulate abnormally in endometriomas or the peritoneal cavity. Endometriosis cells are particularly susceptible to DNA damage due to direct exposure, leading to the formation of reactive oxygen species (ROS). Cells typically respond to genotoxic DNA damage by initiating DNA damage responses to avoid mitosis and facilitate DNA repair. However, excessive oxidative stress, coupled with inadequate repair mechanisms, can lead to endometriosis cell death^29^.

Regarding the distribution of endometriosis stages according to AAGL, the highest proportion was observed in stage IV, comprising 29 participants (52.7%). The AAGL classification system categorizes endometriosis based on the complexity of surgery. Findings from Abrao et al. (2021) corroborate this distribution, reporting that among 1224 patients, 230 individuals (18.79%) had stage I, 321 (26.22%) had stage II, 148 (12.09%) had stage III, and 525 (42.89%) had stage IV endometriosis according to the AAGL classification^25^.

### Differences in ferritin and GPx4 levels based on endometrioma severity according to AAGL stage

Based on Table 4 here was no statistically significant difference observed in the comparison of ferritin and GPx4 levels based on the severity of endometriosis according to the AAGL system (*p* > 0.05). Endometriosis scoring system according to AAGL is based on anatomy and complexity of surgery. The AAGL system classifies endometriosis based on the anatomy and complexity of surgery, considering the involvement of various organs such as the vagina, ureters, fallopian tubes, douglas cavity, intestine, rectovaginal septum, retrocervix, bladder, ovary, small intestine/caecum, and appendix. Each stage is assigned a score based on the total involvement of these organs, with stage I having a total score of ≤ 8, stage II scoring between 9 and 15, stage III between 16 and 21, and stage IV having a score greater than 21^25^.

In this study the samples taken were only from the lining of the endometrioma (in the ovary) selected from the densest chocolate density. Followed by examination of ferritin and GPx4 levels by ELISA. Ferritin and GPx4 levels were not measured for all findings of endometriosis lesions in other organs so that they did not describe the condition of ferritin and GPx4 levels as a whole.

Abrao et al. (2021) conducted a study of endometriosis classification based on the AAGL system from 1224 patients, 230 respondents (18.79%) had stage I, 321 respondents (26.22%) had stage II, 148 respondents (12.09%) had stage III. and stage IV as many as 525 respondents (42.89%). The AAGL system is based on the complexity of the surgery^25^.

### Correlation of ferritin and GPx4 levels

The Table 3; Fig. 1 showed a correlation of ferritin and GPx4 levels in endometrioma tissue. The study revealed a statistically significant negative correlation (*p* < 0.05) between ferritin levels and GPx4 levels, with a correlation coefficient of *r* = -0.600 (p value < 0.001), indicating a highly significant and strong correlation. This finding suggests that in patients with endometrioma, higher levels of ferritin are associated with lower levels of GPx4, potentially leading to ferroptosis within endometriomas.

## Limitations

One of the major limitations of this study is the small sample size, which may affect the generalizability of the findings. Future studies with larger sample sizes are necessary to validate our results and provide more robust conclusions regarding the correlation of ferritin and GPx4 levels as markers of the ferroptosis process in endometrioma.

## Conclusion

In conclusion showed that the ferritin and GPx4 has a significant negative correlation as the biomarker of ferroptosis in endometrioma.

## Data Availability

The datasets used and/or analyzed during the current study are available from the corresponding author on reasonable request.

## Declarations

### Registration of research

The research has been according with guidelines aligned with the Declaration of Helsinki. Patients provided written informed consent for the use of their tissue samples in this research. Ethical clearance was obtained from the Health Research Ethics Committee of Universitas Padjadjaran, registered under LB.02.01/X.6.5/168/2023. All data was registered at researchregistry with Research Registry UIN researchregistry10061.
